# Lipid Goal Achievement With Statins Among Statin-Naïve Indian Patients Undergoing Percutaneous Coronary Intervention

**DOI:** 10.1016/j.jscai.2025.104163

**Published:** 2026-02-24

**Authors:** Mahidhar Jeedigunta, Vivek Veeram Reddy, Ganesh Paramasivam

**Affiliations:** Department of Cardiology, Kasturba Medical College, Manipal Academy of Higher Education, Manipal, India

**Keywords:** lipid goals, lipid-lowering, low-density lipoprotein cholesterol, statin therapy, statin-naïve Indian patients

## Abstract

**Background:**

The effectiveness of current statin therapy in achieving lipid targets in Indian patients remains uncertain. This observational study aimed to evaluate lipid goal attainment at 1 and 6 months with atorvastatin and rosuvastatin in statin-naïve South Indian patients undergoing percutaneous coronary intervention (PCI).

**Methods:**

This prospective, observational, single-center study included 491 statin-naïve patients who underwent PCI between March 2021 and May 2023. Effects of statins on low-density lipoprotein cholesterol (LDL-C) reduction and lipid goal achievement were assessed at 1 and 6 months. Secondary objectives compared the individual efficacy of atorvastatin vs rosuvastatin, the impact of baseline lipids, statin switching, and the association between lipid goal achievement and major adverse cardiovascular events.

**Results:**

Among 491 patients (mean age, 58.7 years), 437 (89%) presented with acute coronary syndrome. Atorvastatin was prescribed to 327 (66%) and rosuvastatin to 164 (34%). Baseline LDL-C was lower in the rosuvastatin group (122.7 ± 46.2 vs 138.2 ± 48.5 mg/dL; *P* = .001). At 1 month, 41% achieved American Heart Association/American College of Cardiology lipid goals (47.6% with rosuvastatin vs 37.1% with atorvastatin; *P* = .041). Atorvastatin to rosuvastatin switching was done in 113 (34.6%) patients. By 6 months, 42.5% achieved goals. Rosuvastatin showed better LDL-C goal attainment (62.8% vs 38.2%; *P* < .001). Rosuvastatin switching modestly improved outcomes (*P* ≤ .036).

**Conclusions:**

Only 41.1% and 42.5% of statin-naïve Indian patients undergoing PCI achieved lipid goals on statins at 1 and 6 months, suggesting a need for more intensive lipid-lowering strategies post-PCI. Rosuvastatin was marginally more efficacious than atorvastatin.

## Introduction

Coronary artery disease (CAD) is the leading cause of death worldwide. In India as well, cardiovascular diseases are the prime cause of mortality.^1^ Dyslipidemia is one of the major risk factors for CAD. Elevated levels of low-density lipoprotein cholesterol (LDL-C) and non–high-density lipoproteins are the major components of dyslipidemia associated with CAD. In India, the dyslipidemia pattern is often termed “atherogenic dyslipidemia,” characterized by elevated serum triglycerides (TG), small dense low-density lipoprotein (LDL) particles, and low levels of high-density lipoprotein (HDL) cholesterol. Most cardiologists and physicians currently target an LDL-C level <70 mg/dL following a CAD event. However, there is a growing trend toward targeting even lower LDL-C levels, with no defined lower limits.^2^

Major studies have shown that LDL-C levels 1 month post–percutaneous coronary intervention (PCI) in acute coronary syndrome (ACS) patients predict the recurrence of coronary events, with lower LDL-C levels being associated with better outcomes.^3^ Because LDL-C plays a key role in the pathogenesis of atherosclerotic cardiovascular disease (ASCVD) and is shown to reduce all-cause mortality, it is rational that LDL level is lowered as much as feasible. Furthermore, the availability of several non-statin agents has enabled us to reduce LDL-C to lower targets. The progressively decreasing recommended LDL-C thresholds set by various international guidelines reflect this trend. Per the American Heart Association (AHA)/American College of Cardiology (ACC) guidelines, patients with ACS should receive high-intensity statin therapy to reduce LDL-C level by ≥50% or to a target of ≤70 mg/dL.^4^ The goals are even more stringent in the Lipid Association of India (LAI) guidelines, wherein an “extremely high risk” group has been described. The LAI recommends an LDL-C goal of <70 mg/dL for very high risk patients and <55 mg/dL for the extremely high risk group.^5^ The level set by the LAI for the extremely high risk group currently suffers from a lack of good randomized trials in our Indian population, with its unique patterns of dyslipidemia and glucose intolerance.^6^

In addition to this specific dyslipidemia pattern, the response to lipid-lowering therapies may vary, as both lipid profiles and drug responses are influenced by genetic factors. The heterogeneity in culture, food habits, and health behaviors in the Indian population makes generalization in these aspects of lipid therapy difficult. Hence, studies catering to knowing the lipid parameters and the response to the medications targeting especially the Indian population at large, as well as regionally, are the need of the hour.^7^, ^8^, ^9^

## Materials and methods

The current study is a prospective, observational, single-center study done at Kasturba Medical College, Manipal, for a period of 2 years from March 2021 to May 2023. The study was approved by the Kasturba Medical College and Kasturba Hospital Institutional Ethics Committee (766/2020) and by the Clinical Trial Registry of India (CTRI) (CTRI/2021/03/031826). Patients aged >18 years with a confirmed diagnosis of ischemic heart disease (IHD) who were naïve to statins and underwent a subsequent PCI were included in this study. Any unstable patients, pregnant patients or patients who died in-hospital during index admission were excluded. A total of 620 patients were initially included in the study, of whom, 491 were finally included after excluding loss to follow-up.

### Description of the process

All admitted patients with IHD undergoing percutaneous revascularization at Kasturba Medical College Hospital, Manipal, who fulfilled the inclusion criteria were enrolled after obtaining informed consent. Details regarding clinical presentation, including clinical history, findings on physical examination, investigations including biochemical tests, electrocardiogram, echocardiography, medical and interventional treatments, were recorded. Baseline LDL-C values were documented, and the same patients were followed up for 1 month and 6 months for routine lipid profile investigations. Dyslipidemia was defined as LDL-C ≥130 mg/dL, non-HDL ≥160 mg/dL, HDL in men <40 mg/dL, HDL in women <50 mg/dL, and serum TG >150 mg/dL.

The primary objective of this study was to compare LDL-C reduction and lipid goal achievement between atorvastatin (40 mg) and rosuvastatin (20 mg) at 1 and 6 months of therapy among statin-naïve patients initiated on these drugs following percutaneous revascularization for IHD. The secondary objectives were to assess the comparative LDL-C–lowering efficacy of atorvastatin (40 mg) and rosuvastatin (20 mg) at 1- and 6-month follow-up and to evaluate the association between baseline lipid levels, attainment of lipid goals, and the occurrence of major adverse cardiovascular events (MACE), including all-cause death, rehospitalization (for all causes and cardiovascular causes), nonfatal myocardial infarction (MI), nonfatal stroke, heart failure and repeat revascularization, over a 6-month follow-up period.

The target LDL-C goals aimed in this study are taken according to AHA/ACC guidelines, which advocate that patients with ACS should receive a high-intensity statin therapy with a goal to reduce LDL-C level by ≥50% or to a target of ≤70 mg/dL (this cut off would be determined by the risk group to which the patient belongs). Similarly, the non-HDL level is targeted to be reduced to <130 mg/dL.^4^ The same was adopted for statistical analysis. The efficacy of statins and adequacy of the therapy were evaluated based on the ability of the drug to achieve the above-mentioned levels of LDL-C.

In-hospital outcomes, including mortality and major adverse cardiovascular events (MACE), were recorded during the index hospitalization. MACE included all-cause death, rehospitalization (for all causes and cardiovascular causes), nonfatal myocardial infarction (MI), nonfatal stroke, heart failure, and repeat revascularization.

Postdischarge follow-up data for 1 and 6 months duration were also obtained during the patient’s routine follow-up visits. In case the patient failed to follow-up, he or she was contacted telephonically to obtain follow-up data.

### Statistical analysis

Data cleaning and analysis were performed using Excel (Microsoft) and SPSS version 22 (IBM Corp), respectively. For categorical data, the χ^2^ test and Fisher exact test were employed, and for continuous data, mean ± SD and *t* test were used to determine significance. Univariable and multivariable logistic regression analysis were done to determine the factors associated with the achievement of lipid goals. A *P* value <.05 was taken as an indicator of statistical significance.

## Results

**Figure 1:**
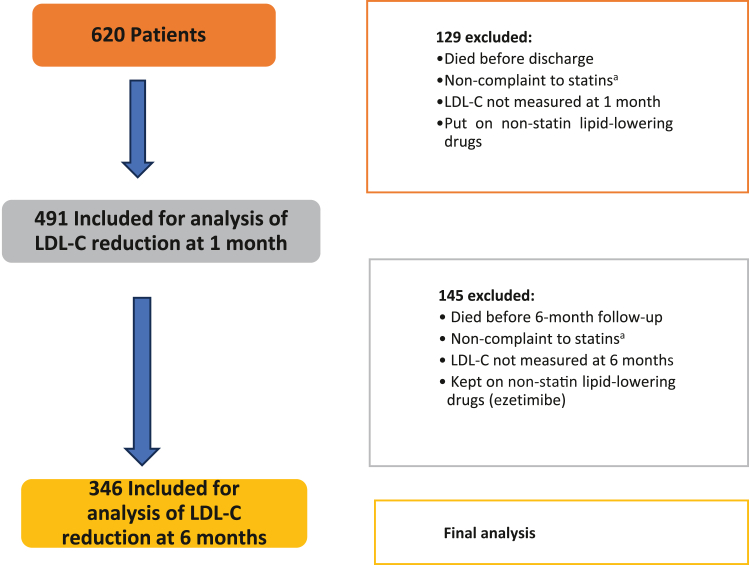
**Study flowchart.** LDL-C, low-density lipoprotein cholesterol. ^a^Non-compliance due to patient-related factors other than drug-related adverse events (personal, logistic, financial, etc).

This study included 620 patients who were admitted to the cardiology department and underwent revascularization, out of whom 129 patients were excluded from the analysis for not meeting the inclusion criteria or being lost to follow-up. A total of 491 patients were included in the analysis at the 1-month follow-up, and a total of 346 patients were included in the final analysis at the 6-month follow-up (Figure 1).

In the study population, the mean age was 58.7 ± 10.9 years, with women constituting 22% (108) of the population. Mean body mass index (BMI) was 24.5 ± 3.2 kg/m^2^, showing that the majority were either overweight or obese (a South Asian cut off of ≥ 27.5 kg/m^2^ for obese and a BMI of 23 to 27.4 kg/m^2^ as overweight, as given by WHO in 2014 was used). A total of 221 (45.0%) patients had diabetes, 258 (52.5%) had hypertension, 436 (88.8%) patients had dyslipidemia, and 22 (4.5%) patients had chronic kidney disease (CKD). Family history of heart disease was seen in 38 (7.7%) patients. A history of smoking and obesity was present in 57 (11.6%) and 189 (38.5%) patients, respectively (Table 1). Median duration of hospital stay was 4 days. The clinical and laboratory parameters at baseline in the study population are depicted in Table 1.

**Table 1:** Baseline clinical and laboratory variables.

Characteristics	N = 491
Age, y	58.7 ± 10.9
Female sex	108 (22%)
Height, m	1.66 ± 0.08
Weight, kg	67.3 ± 9.3
Body mass index, kg/m^2^	24.5 ± 3.2
Risk factors	
Diabetes mellitus	221 (45%)
Hypertension	258 (52.5%)
Chronic kidney disease	
On history	22 (4.5%)
By eGFR	64 (13%)
Tobacco use	57 (11.6%)
Peripheral vascular disease	2 (0.4%)
Cerebrovascular disease	4 (0.8%)
Family history of CAD	38 (7.7%)
Obesity	189 (38.5%)
Dyslipidemia^a^	436 (88.8%)
Creatinine, mg/dL	1.0 ± 0.3
Hemoglobin, g/dL	13.9 ± 2.1
Albumin, g/d	4.4 ± 0.5
Urea, mg/dL	24.3 ±11.8
Thyroid-stimulating hormone, μIU/mL	2.7 ± 3.3
Fasting blood sugar, mg/dL	153.4 ± 70.7
HbA1c, %	6.8 ± 1.7
Sodium, mmol/L	135.8 ± 3.7
Potassium, mmol/L	4.4 ± 0.6
Troponin T, ng/mL	0.237 (0.0415-0.767)
NT-proBNP, pg/mL	433 (122-1509)
Total cholesterol, mg/dL	195.8 ± 54.2
Triglycerides, mg/dL	151.6 ± 87.2
LDL-C, mg/dL	133.0 ± 48.3
HDL-C, mg/dL	40.5 ± 11.2
Non-HDL-C, mg/dL	155 ± 52.7

Values are mean ± SD, n (%), or median (IQR).

CAD, coronary artery disease; HbA1c, glycated hemoglobin; HDL-C, high-density lipoprotein cholesterol; LDL-C, low-density lipoprotein cholesterol.

a Dyslipidemia was defined as LDL-C ≥130 mg/dL, non-HDL ≥160 mg/dL, HDL in men <40 mg/dL, HDL in women <50 mg/dL, and serum triglycerides >150 mg/dL.

In our study, 45.8% of the subjects were classified as being at very high risk of ASCVD events (Table 2).^10^ At baseline, 88.8% of the subjects had dyslipidemia, 62.1% had high LDL-C, 57.2% had high non-HDL-C, 59.5% had low HDL-C and 38.5% had high TG.

**Table 2:** Risk stratification of the study population for guideline-directed therapy.

Major ASCVD events	N = 491
Recent ACS (within the past 12 mo, including index event)	491 (100%)
History of MI (other than the recent ACS event listed above)	0
History of ischemic stroke	4 (0.8%)
Symptomatic PAD^b^	2 (0.4%)
High-risk conditions	
Age >65 y	139 (28.3%)
Diabetes mellitus	221 (45%)
Hypertension	258 (52.5%)
Chronic kidney disease^c^	64 (13.0%)
Smoking	57 (11.6 %)
Heterozygous familial hypercholesterolemia	0
History of prior coronary artery bypass surgery or percutaneous coronary intervention outside of the major ASCVD event(s)	0
Persistently elevated LDL-C (LDL-C ≥100 mg/dL despite maximally tolerated statin therapy and ezetimibe	0
History of congestive HF	0
Extremely high ASCVD risk^a^	225 (45.8%)
Not very high ASCVD risk	266 (54.2%)

ACS, acute coronary syndrome; ASCVD; atherosclerotic cardiovascular disease; HF, heart failure; LDL-C, low-density lipoprotein cholesterol; MI, myocardial infarction; PAD, Peripheral Arterial Disease.

a Extremely high risk includes a history of multiple (>1) major ASCVD events or 1 major ASCVD event and multiple (>1) high risk conditions.^10^

b History of claudication with an ankle-brachial index <0.85 or previous revascularization or amputation.

c Estimated glomerular filtration rate 15-59 mL/min/1.73 m^2^

The majority of our study population (89%) presented with MI, of which 68.2% were ST-segment elevation MI, 20.8% were non–ST-segment elevation MI, whereas 9% were unstable angina and 2% were chronic stable angina. Of these, 276 (56.2%) had preserved ejection fraction (EF), 176 (35.8%) had mid-range EF, and 39 (7.9%) had reduced EF.

Patients with early-onset CAD (aged ≤45 years) were 11.6% in our study. They had a higher median BMI as compared to the rest of the population. In addition, they had a significantly higher LDL-C (152.2 ± 55.3 mg/dL, *P* = .001) and non-HDL-C (176.2 ± 59.8 mg/dL, *P* = .001) at baseline, compared to patients aged >45 years.

Baseline levels of LDL-C, non-HDL-C, and TG showed no significant difference among the gender distribution. However, the prevalence of low HDL-C at baseline (*P* = .017) was higher in women in our study population.

In our study, diabetics, at baseline, had significantly higher prevalence of high LDL-C (69.2% vs 56.3%, *P* = .003), high non-HDL-C (66.5% vs 49.6%, *P* < .001) and high TG (45.2% vs 33.0%, *P* = .005) compared to non-diabetics. Diabetic men had a significantly higher prevalence of low HDL-C at baseline than non-diabetic men (60.8% vs 53.5%, *P* = .031).

Hypertensive patients had a higher incidence of high TG (42.6% vs 33.9%, *P* = .047) and lower LDL-C levels (128.6 ± 48.2 vs 140.0 ± 47.9, *P* = .032) compared to normotensive patients.

### Statin and antiplatelet therapy in the study population

On discharge, all the patients were prescribed aspirin (75 mg once daily after a 325 mg loading dose) as the first antiplatelet and the majority (97.6%) were discharged on ticagrelor (90 mg twice daily after a 180 mg loading dose) as the second antiplatelet. On 1-month follow-up, all the patients were prescribed aspirin as the first antiplatelet and the majority (70.9%) were continued on ticagrelor as the second antiplatelet. At 6 months follow-up, most (99.4%) of the patients were prescribed aspirin (75 mg once daily) as the first antiplatelet and the majority (86.2%) were switched over from ticagrelor (90 mg twice daily) to clopidogrel (75 mg once daily after a 300 mg loading dose) as the second antiplatelet.

In our study, 66.6% patients were discharged on atorvastatin (40 mg), whereas 33.4% patients were discharged on rosuvastatin (20 mg). Of the patients, 94.5% were discharged on high-dose statins (atorvastatin 40 mg or rosuvastatin 20 mg), out of whom 11 (2.2%) were discharged on rosuvastatin 40 mg.

At the 1-month follow-up, 43.8% were continued on atorvastatin 40 mg, whereas 56.2% were continued on rosuvastatin 20 mg. Of the 327 patients (two-thirds of the study population) who were discharged on atorvastatin 40 mg, at the 1-month follow-up, 214 (65.4%) were continued with atorvastatin 40 mg and 113 (34.6%) were switched over to rosuvastatin 20 mg. Out of 164 patients (one-third of the study population) discharged on rosuvastatin 20 mg, at 1 month, 163 (99.4%) were continued on rosuvastatin 20 mg and 1 (0.6%) patient was switched over to atorvastatin. At the 1-month follow-up, the switch over from atorvastatin to rosuvastatin was statistically significant (34.6%, *P* < .001).

At the 6-month follow-up, 19.3% were continuing on atorvastatin (40 mg), whereas 80.6% were receiving rosuvastatin (20 mg).

At any point in time, a higher percentage of patients had achieved non-HDL-C goal compared to those who had achieved the LDL-C goal.

### Lipid goals at 1 month and statins

A higher percentage of patients on rosuvastatin achieved lipid goals (47.6% vs 37.1%, *P* = .041) and LDL-C goals (62.8% vs 38.2%, *P* < .001) compared to atorvastatin (Table 3).

**Table 3:** Patients who achieved target lipid levels on statin therapy.

Target	1 mo	6 mo	Atorvastatin 40 mg at 1 mo (n = 327)	Rosuvastatin 20 mg at 1 mo (n = 164)	*P* value^a^
Lipid goal^b^	202 (41.1%)	147 (42.5%)	124 (37.1%)	78 (47.6%)	.041
LDL-C goal	228 (46.4%)	173 (50.0%)	125 (38.2%)	103 (62.8%)	<.001
Non–HDL-C goal	315 (64.2%)	217 (62.7%)	196 (59.9%)	119 (72.6%)	.006
≥50% reduction in LDL-C	245 (49.9%)	174 (50.3%)	161 (49.2%)	84 (51.2%)	.678
LDL-C <70 mg/dL	296 (60.3%)	199 (57.5%)	175 (53.5%)	121 (73.8%)	<.001
LDL-C <55 mg/dL	151 (30.8%)	113 (32.7%)	72 (22.0%)	79 (48.2%)	<.001
LDL-C <30 mg/dL	10 (2.03%)	9 (2.6%)	1 (0.3%)	9 (5.4%)	**–**

HDL-C, high-density lipoprotein cholesterol; LDL-C, low-density lipoprotein cholesterol.

a *P* value calculated is for atorvastatin/rosuvastatin at 1 mo.

b Reduction of LDL-C by ≥50% or to a target of ≤70 mg/dL and non-HDL reduction to <130 mg/dL, as per the AHA/ACC guidelines.

Target LDL-C <70 mg/dL and <55 mg/dL at 1 month was achieved more with rosuvastatin compared to atorvastatin (73.8% vs 53.5%, *P* < .001) and (48.2% vs 22.0%, *P* < .001), respectively. Non-HDL-C goals were achieved in a significantly higher percentage of patients on rosuvastatin compared to atorvastatin (72.6% vs 59.9%, *P* = .006) (Table 3).

There was a significant difference noted in the mean baseline levels of LDL-C (138.2 ± 48.5 vs 122.7 ± 46.2 mg/dL, *P* = .001), non-HDL-C (160.2 ± 52.7 vs 145.2 ± 51.3 mg/dL, *P* = .003), and total cholesterol (201.3 ± 54.1 vs 184.9 ± 53.0 mg/dL, *P* = .002) between the atorvastatin and rosuvastatin groups, respectively. Patients in the rosuvastatin group had significantly lower levels at baseline (Table 4).

**Table 4:** Comparison of mean lipid levels at the baseline and 1-month follow-up and response to statins.

Variable	Atorvastatin 40 mg	Rosuvastatin 20 mg	*P* value
LDL-C at baseline, mg/dL	138.2 ± 48.5	122.7 ± 46.2	.001
LDL-C at 1 mo, mg/dL	72.5 ± 27.2	60.9 ± 29.5	<.001
Non-HDL-C at baseline, mg/dL	160.2 ± 52.7	145.2 ± 51.3	.003
Non-HDL-C at 1 mo, mg/dL	92.0 ± 30.2	84.3 ± 33.5	.01
HDL-C at baseline, mg/dL	41.0 ± 11.1	39.7 ± 11.1	.206
HDL-C at 1 mo, mg/dL	39.8 ± 9.6	40.0 ± 10.6	.825
Triglycerides at baseline, mg/dL	151.6 ± 83.0	151.6 ± 95.2	.997
Triglycerides at 1 mo, mg/dL	137.6 ± 60.5	143.8 ± 72.5	.316
Total cholesterol at baseline, mg/dL	201.3 ± 54.1	184.9 ± 53.0	.002
Total cholesterol at 1 mo, mg/dL	131.8 ± 31.5	124.3 ± 34.8	.016

HDL-C, high-density lipoprotein cholesterol; LDL-C, low-density lipoprotein cholesterol.

At the 1-month follow-up, mean levels of LDL-C (72.5 ± 27.2 vs 60.9 ± 29.5 mg/dL, *P* < .001), non-HDL-C (92.0 ± 30.2 vs 84.3 ± 33.5 mg/dL, *P* = .01), and total cholesterol (131.8 ± 31.5 vs 124.3 ± 34.8 mg/dL, *P* = .016) were lower and statistically significant in the rosuvastatin group in comparison to the atorvastatin group (Table 4).

A higher percentage of diabetics achieved LDL-C <70 mg/dL and LDL-C <55 mg/dL compared to non-diabetics (*P* = .002), although overall lipid or LDL-C goals showed no significant difference between the 2 groups.

This may be attributed to the statistically significant lower mean LDL-C level at baseline (127.6 ± 46.1 vs 137.6 ± 49.6 mg/dL, *P* = .022) in diabetics compared to nondiabetics.

Diabetics also had higher mean levels of TG at baseline (137.5 ± 68.8 vs 168.8 ± 102.9 mg/dL, *P* < .001), in comparison with non-diabetics.

Diabetics on rosuvastatin achieved significantly better lipid goals (57.0% vs 38.8%, *P* = .020), LDL-C <70 mg/dL (81.0% vs 67.1%, *P* = .042), and LDL-C <55 mg/dL (60.8% vs 36.5%, *P* = .002), respectively, compared to non-diabetics.

Patients on atorvastatin at discharge with diabetes had statistically significantly lower mean LDL-C level at baseline compared to non-diabetics (130.7 ± 46.1 vs 144.0 ± 49.6 mg/dL, *P* = .013).

There was no difference between mean LDL-C levels at baseline for patients on rosuvastatin with or without diabetes. However, diabetics on rosuvastatin had statistically significantly lower mean LDL-C levels on 1-month follow-up (55.8 ± 34.2 vs 65.6 ± 23.5 mg/dL, *P* = .018). Patients with CKD did not achieve lipid goals (defined above) as compared to patients without CKD (*P* = .011).

### Lipid goals at 1-month: Multivariable analysis

Multivariable analysis for factors associated with achievement of lipid goals at 1 month was assessed as shown in Table 5, in which we have analyzed baseline LDL-C levels as the first variable.

**Table 5:** Factors associated with achievement of lipid goals at 1 month.

Variables	Univariable		Multivariable	
	Odds ratio (95% CI)	*P* value	Odds ratio (95% CI)	*P* value
Baseline LDL-C level	1.009 (1.005-1.013)	<.001	1.010 (1.006-1.015)	<.001
Rosuvastatin 20 mg at discharge	1.485 (1.016-2.170)	.041	1.716 (1.149-2.565)	.008
Age	0.996 (0.979-1.012)	.609	–	–
Female sex	1.133 (0.736-1.745)	.570	–	–
Diabetes	1.272 (0.886-1.825)	.192	1.357 (0.928-1.986)	.116
Chronic kidney disease	0.473 (0.263-0.851)	.012	0.548 (0.299-1.005)	.052
Tobacco use	0.520 (0.283-0.956)	.035	0.564 (0.300-1.058)	.074
Obesity	0.939 (0.649-1.360)	.741	–	–

LDL-C, low-density lipoprotein cholesterol.

Baseline LDL-C level (*P* < .001) and rosuvastatin at discharge (*P* = .008) seem to significantly affect the achievement of lipid goals in our study population.

## Analysis of lipid goals at 6 months

A total of 346 patients who were followed up at 6 months with a lipid profile were analyzed separately. Among them, the mean age was 58.2 ± 10.9 years, mean BMI was 24.7 ± 3.2 kg/m^2^, median duration of hospital stay was 4 days, mean baseline LDL-C was 132.4 ± 48.3 mg/dL, mean non-HDL-C was 155.3 ± 52.7 mg/dL, mean HDL-C was 41.0 ± 11.3 mg/dL, mean TG were 150.7 ± 85.0 mg/dL, and mean total cholesterol was 196.3 ± 54.5 mg/dL; women were 19.7% of the analyzed study population, 43.1% were diabetics, 50.6% were hypertensive, 13% were tobacco users, and 41.3% were obese. This population had similar baseline characteristics as our study population of 491 patients that were analyzed at 1 month.

A significant number of patients were switched over from atorvastatin to rosuvastatin at 1-month follow-up, in accordance with our institute protocol. At 1 month, 43.8% were prescribed atorvastatin, whereas 56.2% were prescribed rosuvastatin.

The switch over from atorvastatin to rosuvastatin was significant (*P* < .001).

When we analyzed whether patients were switched from atorvastatin to rosuvastatin because of not achieving lipid goals, it was found to be non-significant, and the switch to rosuvastatin at 1 month was as per the institute protocol.

Lipid goals were better achieved in patients who were prescribed or switched over to rosuvastatin at 1-month follow-up (53.3% vs 39.9%, *P* = .043).

Of the 491 patients, 10 patients achieved ≤30 mg/dL LDL-C at 1 month. Of these, one patient was receiving atorvastatin 40 mg, and 9 patients were receiving rosuvastatin 20 mg. Similarly, at the end of 6 months, 9 patients had LDL-C ≤30 mg/dL, of which 1 patient was on atorvastatin 40 mg, and 8 patients were on rosuvastatin 20 mg.

### Lipid goals at 6 months: Multivariable analysis

Multivariable analysis for factors associated with achievement of lipid goals at 6 months was performed as described in Table 6, wherein we analyzed baseline LDL-C levels as the first variable.

**Table 6:** Factors associated with achievement of lipid goals at 6 months.

Variables	Univariable analysis			Multivariable analysis		
	OR	95% CI	*P* value	OR	95% CI	*P* value
Baseline LDL-C level	1.013	1.008-1.018	<.001	1.013	1.008-1.018	<.001
Rosuvastatin at discharge^a^	0.711	0.445-1.138	.155	0.993	0.573-1.723	.981
Switch from A to R at 1 mo	1.719	1.016-2.907	.043	1.773	1.011-3.109	.046
Age	0.994	0.975-1.014	.537	–	–	–
Female sex	1.460	0.858-2.486	.163	–	–	–
Diabetes	1.254	0.816-1.928	.303	–	–	–
Chronic kidney disease	0.353	0.163-0.768	.009	0.395	0.173-0.903	.005
Tobacco use	0.342	0.163-0.716	.004	0.330	0.152-0.903	<.001
Obesity	0.918	0.595-1.416	.698	–	–	–

A, atorvastatin; LDL-C, low-density lipoprotein cholesterol; OR, odds ratio; R, rosuvastatin.

a Rosuvastatin 20 mg.

Baseline LDL-C level (*P* < .001), statin switch from atorvastatin to rosuvastatin at 1 month (*P* = .046), CKD (*P* = .005), and tobacco usage (*P* ≤ .001) seem to significantly affect the achievement of lipid goals in our study population (Table 6).

Of the 491 patients, 18 (3.7%) reported having MACE. Univariable analysis for MACE showed a significant association of LDL-C, non-HDL-C, female sex, and left main coronary artery disease with MACE.

However, only left main coronary artery disease showed a significant association with MACE in multivariable analysis (*P* = .008).

The lipid goals at 1 month did not significantly affect MACE. This may be attributed to the smaller event rate and the relatively short follow-up period in our study.

Long-term follow-up is required to assess whether lipid goals translate to affect MACE in our study.

## Discussion

Indians and Asians have been shown to have an earlier onset of cardiovascular events in addition to a more aggressive progression of ASCVD. Moreover, most studies from the subcontinent have shown that Indians have atherogenic dyslipidemia with not only high levels of LDL-C but also low levels of HDL-C and hypertriglyceridemia.^6^^,^^11^, ^12^, ^13^

This translates into a hurdle when faced with the acute necessity of controlling the blood cholesterol levels in a short gap, as in, after an ACS. Failure to control the LDL-C levels to the values set in lipid guidelines of multiple societies leads to recurrence of ischemia and death, which are particularly high in the early months of an ACS event.^14^

The main objective of this observational study was to evaluate the lipid parameters in patients given secondary prevention with statins and the achievement of lipid goals. Statins, for the last 30 years, have been the sheet anchor of lipid-lowering therapy, with an excellent efficacy and safety profile. However, in the Indian scenario, the issue of concern is the fact that 60% of the patients post-revascularization fail to achieve either of the target lipid goals. And with the changing scenario where multiple affordable lipid-lowering non-statin drugs are available in the market, the control of lipid parameters post-ACS or post-revascularization is important.

This observational study demonstrated that in a cohort of post-ACS patients or patients undergoing revascularization, only 202 (41.1%) of the subjects achieved lipid goals at 1 month, and despite adequate change of statin dose and drug, only 147 (42.5%) achieved the LDL-C goals at 6 months.

Multiple Indian studies in this aspect have shown that in majority of the cases, even despite high-intensity statin therapy, Indian patients fail to achieve the lipid goals. All the studies in this manner have shown failure to reduce LDL-C levels, especially in the early months (Supplemental Table S1).

In a study by Jain et al,^15^ which is a multicenter observational study across 11 centers, it was found that 55.6% of the 575 patients were unable to meet the lipid goals post-revascularization.

Availability of non-statin options that act synergistically to statins, such as combination therapy with ezetimibe, bempedoic acid, PCSK9 inhibitors, and inclisiran, is an exciting avenue currently shown in majority of the studies to reduce LDL-C to guideline-directed levels.^16^ However, the impact of reduction on short- and long-term morbidity and mortality currently is not clear.

In the retrospective observational study by Bansal et al,^17^ in the subset of patients on high-intensity statin therapy alone, only one-third of the patients achieved the lipid goals. Likewise, in the Optimization of LDL-C in the Stable CAD study done by Mahajan et al,^18^ which observed the lipid-lowering therapy and lipid targets in Indian patients with angiographically proven CAD, only 25.9% achieved the LAI advocated lipid goals of <50 mg/dL and 46.4% achieved AHA lipid goals of <70 mg/dL. In a similar recently published study from Mumbai by Shah et al, among patients with established ASCVD, only 42.16% of the patients achieved lipid targets with rosuvastatin single therapy and another 43.28% achieved the target of <55 mg/dL with rosuvastatin and ezetimibe.^19^

This picture is similar to the scenario worldwide, where the monotherapy with statins, immediate post-ACS, led to the underachievement of lipid goals. For example, the Dyslipidemia International Study II, a Chinese multicenter study showed that, at 6 months of therapy, only 41.2% of the subjects could achieve the LDL-C goals.^20^ Majority of the European studies post-ACS with either statin monotherapy or statin plus ezetimibe therapy have shown inability to achieve lipid goals.

Similarly, in our study, 58.9% of patients on high-intensity statins, either with rosuvastatin or atorvastatin, failed to achieve the target LDL-C at 1 month, and 67.5% failed to achieve the target at 6 months also.

Taking this factor into consideration, the next rationale is to go for upfront combination therapy with all available combination therapies and newer therapies like PCSK9 inhibitors and inclisiran. In the LAI REACT study done with upfront combination therapy with rosuvastatin, ezetimibe, and bempedoic acid started during index admission, 76.3% achieved LAI lipid goals, and 92.2% achieved the ACC lipid goals within 2 weeks, which were found to be sustained at 4 to 6 weeks.^16^

Multiple possible causes for the inability to achieve target lipid goals include lack of starting upfront combination therapy, lack of intensification of therapy at first follow-up, lack of information regarding the genetics and cultural food habits and how they affect the statin efficacy, which may play a possible role in underachieving the lipid goals. Moreover, it has to be borne in mind that statins cause a good reduction of 50% to 60% of LDL-C levels. Hence, even at full dose, if highly effective, they may end up underachieving target LDL-C levels, especially in patients with high baseline LDL-C levels. In addition, majority of the patients are stratified to a very high-risk level, requiring an even lower goal for LDL-C, which is even more difficult. However, similar results worldwide reflect that this is not an exclusive Indian phenomenon, and the upfront therapy with other lipid-lowering drugs in the immediate peri-ACS period needs further validation.

As mentioned in the recent European guidelines, the degree of LDL-C level as well as the duration of exposure plays an important role in the overall risk of ASCVD; upfront combination therapy to rapidly reduce the lipid levels to achieve the target levels is strongly emphasized by multiple guideline committees.^21^^,^^22^

Similarly, genetic single-nucleotide polymorphisms were identified that influence the baseline serum lipid profiles in addition to influencing the response to statins.^23^ However, parallel studies evaluating the genetic basis of dyslipidemic patterns and response to statins are lacking in the Indian population and are currently the need of the hour.

Elevated Lp(a) has been strongly associated with premature and accelerated CAD. Case-control and angiographic studies from various regions of India have shown that higher Lp(a) concentrations correlate with greater coronary plaque burden and earlier onset of MI. In a study on the South Asian population by Joseph et al,^24^ higher Lp(a) levels were significantly associated with an increased likelihood of CAD and the median serum Lp(a) levels were associated with more severe disease, especially in the younger population (aged <50 years). Despite the heterogeneity in assays and reporting Lp(a), the overall evidence supports Lp(a) as a major genetic and independent cardiovascular risk factor in Indians, advocating for routine measurement of Lp(a) levels for better risk stratification and management.^24^, ^25^, ^26^

The presence of CKD and tobacco use significantly reduced the achievement of lipid goals in univariable and multivariable analysis in our study. This is similar to the known picture that shows less benefit of statin therapy in advanced CKD and may be due to multiple risk factors coalescing, leading to more CAD risk in general and worse atherogenic dyslipidemia pattern in particular.^27^^,^^28^

Guideline-recommended LDL-C targets for patients following ACS vary across regions. The 2025 ACC/AHA guidelines advocate for high-intensity statin therapy with a primary LDL-C target of <70 mg/dL for patients at a very high risk, with the addition of non-statin therapies, such as ezetimibe or PCSK9 inhibitors, if this goal is not achieved. In contrast, the 2023 European Society of Cardiology/European Atherosclerosis Society guidelines recommend more stringent targets, with LDL-C <55 mg/dL for very high risk patients and <40 mg/dL for those experiencing recurrent ischemic events within 2 years, alongside a ≥50% reduction from baseline. Indian recommendations, as outlined by the LAI, are even more aggressive, aiming for LDL-C <50 mg/dL in post-ACS patients, with consideration of <30 mg/dL in select high risk cases, emphasizing high-dose statins and combination therapies, including PCSK9 inhibitors when indicated. Collectively, these guidelines underscore a trend toward progressively lower LDL-C targets in very high risk populations, reflecting growing evidence that intensive lipid-lowering strategies confer superior cardiovascular protection.^13^^,^^29^^,^^30^

Recent guideline statements and consensus documents increasingly recognize the value of early combination lipid-lowering therapy for patients at very high cardiovascular risk, particularly after ACS. The LAI suggests initiating combination therapy with a high-intensity statin plus ezetimibe in high risk patients if a >65% reduction in LDL-C levels is anticipated. Similarly, the 2025 focused update of the 2019 European Society of Cardiology/European Atherosclerosis Society dyslipidemia management guidelines advises upfront combination therapy with a statin and ezetimibe in selected high risk, treatment-naïve patients who are unlikely to achieve LDL-C targets with statin monotherapy alone. American society guidelines emphasize high-intensity statins as first-line, but they endorse adding ezetimibe (and other non-statins) promptly when LDL-C goals are unlikely to be met with statin alone and suggest earlier use of combination therapy for very high risk or statin-intolerant patients.^13^^,^^29^^,^^31^

Finally, the persistent clinical inertia in initiating high-intensity statins and adjunctive non-statin therapies for early lipid reduction following ACS appears to stem from multiple factors, foremost among them being inadequate clinician awareness. Addressing this gap requires comprehensive patient-provider education models, implementation of system-level reforms, and a multidisciplinary team–based approach that actively engages patients and their caregivers in post-ACS care. Enhancing clinician education and awareness, streamlining clinical workflows, and integrating electronic health records across specialties are likely to facilitate more consistent adoption of evidence-based lipid-lowering therapy practices.^32^

Our study was a single-center study, and the results may be more applicable to the local populace, although they may be generalizable to a good amount of ethnic Indian and global populations, wherein the data regarding the lipid targets and efficacy of lipid-lowering drugs are still scarce. There was no usage of multiple available oral agents to treat dyslipidemia at 1 month, partly because of lack of guidelines or trial evidence for the same at the start of the study and partly due to the institute policy of gradually upgrading the anti-lipidemic agents. The rosuvastatin dose was not increased beyond 20 mg because of perceived higher incidence of statin-induced myalgias and myopathies in this population, which may have led to the physician underutilization of 40 mg rosuvastatin. And the majority of recruited patients had ACS and were at “very high risk” (45.8%) as per ASCVD risk stratification, which may have affected the efficacy of statins.

## Conclusion

Only 41.1% and 42.5% of statin-naïve Indian patients undergoing PCI achieve their lipid goals at 1 and 6 months, respectively. Rosuvastatin appears to be marginally better than atorvastatin in achieving lipid goals (Central Illustration).

**Central Illustration:**
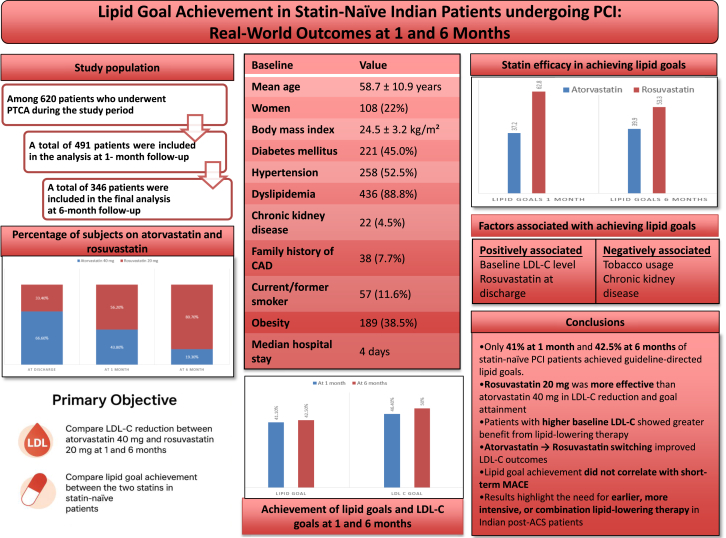
**Summary of the results and conclusion of the study.** ACS, acute coronary syndrome; CAD, coronary artery disease; LDL-C, low-density lipoprotein cholesterol; PCI, percutaneous coronary intervention.

The study shows that despite starting high-intensity statin in statin-naïve patients, the index population fails to achieve the lipid goals (about 63.6%) in the post-revascularization setting, prompting more intensive treatment from the onset in this set of population. Moreover, rosuvastatin is more efficacious in lowering LDL-C and achieving cholesterol goals compared to atorvastatin in statin naïve patients or in patients failing atorvastatin therapy. Patients with higher baseline LDL-C levels tend to benefit more from LDL reduction therapy compared with those having initial lower LDL-C levels. It is also noted from this study that patients with early-onset CAD had higher BMI and higher LDL-C and non-HDL-C levels, patients with diabetes responded better with rosuvastatin therapy, and patients with CKD did not achieve LDL-C levels as expected compared to those with normal renal function.
