# Supplementary material for: Lipid Goal Achievement With Statins Among Statin-Naïve Indian Patients Undergoing Percutaneous Coronary Intervention
Source: J Soc Cardiovasc Angiogr Interv. 2026 Feb 24;5(3):104163. doi: 10.1016/j.jscai.2025.104163 (PMC13005391; doi:10.1016/j.jscai.2025.104163)
Supplement: Supplemental Material [file mmc1.docx]

**Supplemental Table S1. Related studies in Indian context**

| **Study, year** | **Design** | **Results** |
| --- | --- | --- |
| Jain et al, 2023^15^ | 575 patients from 11 centers; high-intensity statins | - 55.65% unable to meet the LDL-C goal. - Only 20.87% patients managed to achieve target LDL-C of <55 mg/dL |
| LAI REACT, 2024^16^ | Used upfront triple therapy with rosuvastatin, ezetimibe, bempedoic acid | - Triple combination regimen was found to be highly efficacious, safe and affordable. - Achieved target LDL-C <50 mg/dL in the first week in 59.6% of patients |
| Bansal et al, 2024^17^ | Patients on high-intensity statin | - One third of patients achieved the lipid goals |
| Mahajan et al, 2024^18^ | Achievement of LAI lipid goals in angiographically proven CAD patients | - 25.9% achieved LAI lipid goals of <50 mg/dL and 46.4% achieved AHA lipid goals of <70 mg/dL |

AHA, American Heart Association; CAD, coronary artery disease; LAI, Lipid Association of India; LDL-C, low-density lipoprotein cholesterol.
